# Crystal Structure of (*E*)-2-(3,3,3-tri­fluoro­prop-1-en-1-yl)aniline

**DOI:** 10.1107/S2056989018012756

**Published:** 2018-09-18

**Authors:** Koji Kubono, Keita Tani, Masaaki Omote, Futa Ogawa, Taisuke Matsumoto

**Affiliations:** a Division of Natural Sciences, Osaka Kyoiku University, Kashiwara, Osaka 582-8582, Japan; b Faculty of Pharmaceutical Sciences, Setsunan University, Hirakata, Osaka 573-0101, Japan; c Institute for Materials Chemistry and Engineering, Kyushu University, Kasuga, Fukuoka 816-8580, Japan

**Keywords:** crystal structure, 3,3,3-tri­fluoro­prop-1-en, aniline, hydrogen bonding

## Abstract

The mol­ecule of the title compound, C_9_H_8_F_3_N, adopts an *E* configuration with respect to the C=C double bond. The dihedral angle between the benzene ring and the prop-1-enyl group is 25.4 (3)°. In the crystal, mol­ecules are linked *via* pairs of N—H⋯F hydrogen bonds into inversion dimers with an *R*_2_^2^(16) ring motif. The dimers are linked by C—H⋯N hydrogen bonds, forming a ribbon structure along the *b-*axis direction. The ribbons are linked by N—H⋯π and C—H⋯π inter­actions, generating a three-dimensional network.

## Abstract 2

The mol­ecule adopts an *E* configuration at the C=C double bond. The dihedral angle between the benzene ring and the prop-1-enyl group is 25.4 (3)°. In the crystal, mol­ecules are linked *via* N—H⋯F hydrogen bonds, forming inversion dimers which are linked into ribbons along the *b* axis by C—H⋯N hydrogen bonds. The ribbons are linked by N—H⋯π and C—H⋯π inter­actions, generating a three-dimensional network.

## Chemical context   

Fluorescein, rhodamine *etc*. are water-soluble fluorescent reagents. Their derivatives exhibit strong fluorescence in aqueous solution and so can be utilized as ion-probes and in bio-imaging (Aron *et al.*, 2016 ▸; Li *et al.*, 2016 ▸). However, complicated procedures are required to obtain them. It is therefore desirable to develop a new fluorescent reagent with a simple structure that can be obtained by a short-step synthetic process. The title compound has a quite simple structure and is a small mol­ecule, consisting of aniline and 3,3,3-tri­fluoro­prop-1-enyl units, which emits strong fluorescence not only in organic solvents but also in an aqueous medium (H_2_O/DMSO, 90:10, *v*/*v*). Since aniline derivatives with 2,4-bis­(3,3,3-tri­fluoro­prop-1-en­yl) have been used as fluoro­genic substrates for dipepeptidyl peptidase-4 (Ogawa *et al.*, 2017 ▸), the title compound can be treated as a simple but essential component in emitting fluorescence. Hence, it is important to study the relationship between the fluorescent properties and the mol­ecular structure of the title compound. We report here its mol­ecular and crystal structure.
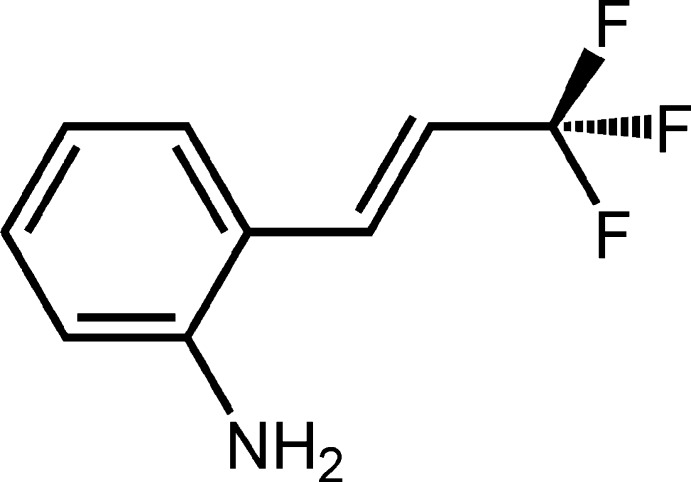


## Structural commentary   

**Figure 1:**
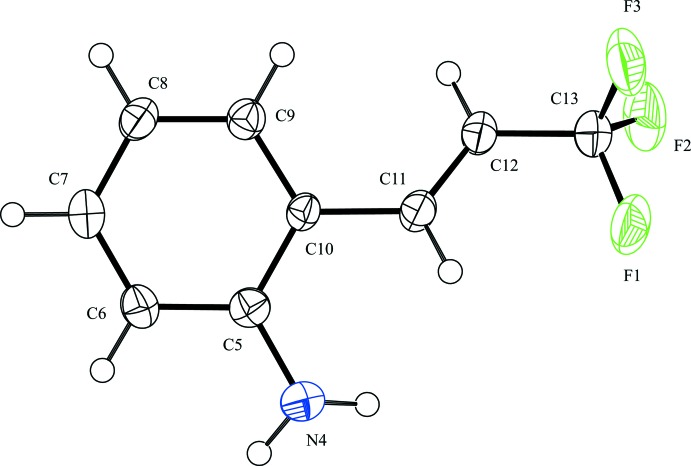
The mol­ecular structure of the title compound with the atom-labelling scheme. Displacement ellipsoids for non-H atoms are drawn at the 50% probability level.

The mol­ecular structure of the title compound is shown in Fig. 1 ▸. The mol­ecule adopts an *E* configuration with respect to the C=C double bond. The dihedral angle between the benzene ring and the prop-1-enyl group is 25.4 (3)°. The C5—C10—C11—C12 and C9—C10—C11—C12 torsion angles are −158.9 (3) and 24.6 (4)°, respectively. The bond lengths and angles in the title compound are normal and agree with those in other tri­fluoro­propenylaniline compounds (Shimizu *et al.*, 2009 ▸; Lin *et al.*, 2014 ▸).

## Supra­molecular features   

**Figure 2:**
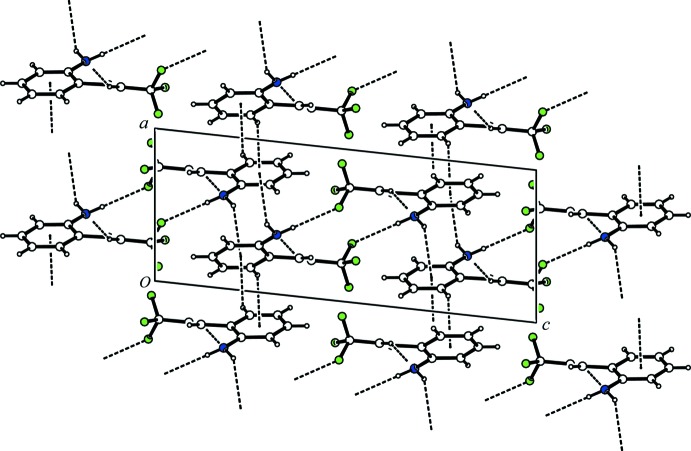
A packing diagram of the title compound, viewed along the *b* axis. The N—H⋯F and C—H⋯N hydrogen bonds and N—H⋯π and C—H⋯π inter­actions are shown as dashed lines.

In the crystal, two mol­ecules are associated through a pair of inter­molecular N—H⋯F hydrogen bonds (Table 1 ▸), forming a centrosymmetric dimer with an 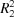
(16) ring motif (Fig. 2 ▸). The dimers are further linked by C—H⋯N hydrogen bonds (Table 1 ▸), forming a ribbon with a *C*(6) chain motif along the *b-*axis direction. The ribbons are linked by N—H⋯π and C—H⋯π inter­actions (Table 1 ▸), generating a three-dimensional network.

## Database survey   

A search of the Cambridge Structural Database (CSD, Version 5.39; May 2018; Groom *et al.*, 2016 ▸) gave 16 hits for 2-(3,3,3-tri­fluoro­prop-1-en-1-yl)aza­benzene derivatives, and gave 18 and 45 hits for (*E*)-3,3,3-tri­fluoro­prop-1-enyl and 2-amino­phenyl-1-enyl fragments, respectively. Of these structures, those that resemble the title compound are 4-[2-(3,3,3-tri­fluoro­prop-1-en-1-yl)phen­yl]morpholine (Lin *et al.*, 2014 ▸), *N*-acetyl-*N*-{2-[(*Z*)-2-chloro-3,3,3-tri­fluoro­prop-1-en­yl]phen­yl}acetamide (Niu *et al.*, 2009 ▸) and (*E*,*E*)-1,4-di­piperidino-2,5-bis­(3,3,3-tri­fluoro­prop-1-en­yl)benzene (Shim­izu *et al.*, 2009 ▸).

## Synthesis and crystallization   

The title compound was prepared by a modification of a reported procedure (Omote *et al.*, 2013 ▸). In a glove box purged with argon gas, iodo­aniline (1.0 mmol), (2-methyl­all­yl)palladium(II) chloride dimer (0.1mmol), CuF_2_ (2.0 mmol) and 2,2′-bipyridyl (2.0 mmol) were placed in a flask. To the flask were added anhydrous DMF (6.0 ml) and (*E*)-trimethyl-(3,3,3-tri­fluoro­prop-1-en­yl)silane (2.0 mmol), and the mixture was stirred at 353 K. After the reaction mixture had been stirred for 4 h, it was poured into ice–water. The mixture was extracted with CH_2_Cl_2_, and the organic layer was dried over anhydrous MgSO_4_. After the solid had been filtered off, the solvent was removed *in vacuo*, and the residue was purified by silica gel column chromatography to give the product in 68% yield. Colourless single crystals were obtained by recrystallization from an ethyl acetate–hexane (1:10, *v*/*v*) solution (m.p. 321–322 K). ^1^H NMR (CDCl_3_) δ: 3.81 (2H, *s*), 6.13 (1H, *qd*, *J* = 15.9, 6.5 Hz), 6.72 (1H, *dd*, *J* = 8.2, 0.9 Hz), 6.80 (1H, *dt*, *J* = 7.5, 0.9 Hz), 7.18 (1H, *dt*, *J* = 7.8, 1.4 Hz), 7.24 (1H, *qd*, *J* = 15.9, 2.1 Hz), 7.29 (1H, *dd*, *J* = 7.8, 1.4 Hz). ^13^C NMR (CDCl_3_) δ: 116.6 (*q*, *J* = 33.4 Hz), 116.8, 119.2, 119.4, 123.6 (*q*, *J* = 269.0 Hz), 127.9, 130.9, 133.3 (*q*, *J* = 6.8 Hz), 144.8. ^19^F NMR (CDCl_3_) δ: 12.07 (3F, *dd*, *J* = 6.5, 2.2 Hz). MS *m*/*z* 187 (*M* ^+^), HRMS calculated for C_9_H_8_F_3_N 187.1617 (*M* ^+^), found 187.0603.

## Refinement   

Crystal data, data collection and structure refinement details are summarized in Table 2 ▸. The amino H atoms were located in a difference Fourier map and refined freely. The C-bound H atoms were positioned geometrically (C—H = 0.93–0.97 Å) and refined using a riding model with *U*_iso_(H) = 1.2*U*_eq_(C). One outlier (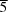
11) was omitted in the last cycle of refinement.

## Supplementary Material

Crystal structure: contains datablock(s) global, I. DOI: 10.1107/S2056989018012756/is5499sup1.cif

Structure factors: contains datablock(s) I. DOI: 10.1107/S2056989018012756/is5499Isup2.hkl

CCDC reference: 1866671

Additional supporting information:  crystallographic information; 3D view; checkCIF report

## Figures and Tables

**Table 1:** Hydrogen-bond geometry (Å, °) *Cg*1 is the centroid of the C5–C10 ring.

*D*—H⋯*A*	*D*—H	H⋯*A*	*D*⋯*A*	*D*—H⋯*A*
N4—H4*A*⋯F2^i^	0.90 (3)	2.46 (4)	3.352 (3)	169 (3)
C12—H12⋯N4^ii^	0.95	2.56	3.432 (4)	152
N4—H4*B*⋯*Cg*1^iii^	0.88 (3)	2.59 (4)	3.315 (2)	140 (3)
C9—H9⋯*Cg*1^iv^	0.95	2.73	3.480 (3)	136

Symmetry codes: (i) 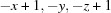; (ii) 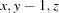; (iii) 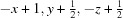; (iv) 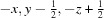.

**Table 2:** Experimental details

Crystal data	
Chemical formula	C_9_H_8_F_3_N
*M*_r_	187.16
Crystal system, space group	Monoclinic, *P*2_1_/*c*
Temperature (K)	123
*a*, *b*, *c* (Å)	7.3925 (4), 6.2777 (3), 18.6065 (9)
β (°)	96.243 (7)
*V* (Å^3^)	858.37 (8)
*Z*	4
Radiation type	Cu *K*α
μ (mm^−1^)	1.16
Crystal size (mm)	0.40 × 0.26 × 0.08

Data collection	
Diffractometer	Rigaku R-AXIS RAPID
Absorption correction	Multi-scan (*ABSCOR*; Higashi, 1995 ▸)
*T*_min_, *T*_max_	0.543, 0.912
No. of measured, independent and observed [*F* ^2^ > 2.0σ(*F* ^2^)] reflections	4753, 1566, 1178
*R*_int_	0.049
(sin θ/λ)_max_ (Å^−1^)	0.602

Refinement	
*R*[*F* ^2^ > 2σ(*F* ^2^)], *wR*(*F* ^2^), *S*	0.061, 0.175, 1.03
No. of reflections	1566
No. of parameters	126
H-atom treatment	H atoms treated by a mixture of independent and constrained refinement
Δρ_max_, Δρ_min_ (e Å^−3^)	0.49, −0.39

Computer programs: *RAPID-AUTO* (Rigaku, 2006 ▸), *SIR92* (Altomare *et al.*, 1993 ▸), *SHELXL2014/7* (Sheldrick, 2015 ▸), *PLATON* (Spek, 2009 ▸) and *CrystalStructure* (Rigaku, 2016 ▸).

## supplementary crystallographic information

### Crystal data

**Table:**

C_9_H_8_F_3_N	*F*(000) = 384.00
*M**_r_* = 187.16	*D*_x_ = 1.448 Mg m^−^^3^
Monoclinic, *P*2_1_/*c*	Cu *K*α radiation, λ = 1.54187 Å
*a* = 7.3925 (4) Å	Cell parameters from 4006 reflections
*b* = 6.2777 (3) Å	θ = 4.8–68.2°
*c* = 18.6065 (9) Å	µ = 1.16 mm^−^^1^
β = 96.243 (7)°	*T* = 123 K
*V* = 858.37 (8) Å^3^	Platelet, colourless
*Z* = 4	0.40 × 0.26 × 0.08 mm

### Data collection

**Table:**

Rigaku R-AXIS RAPID diffractometer	1178 reflections with *F*^2^ > 2.0σ(*F*^2^)
Detector resolution: 10.000 pixels mm^-1^	*R*_int_ = 0.049
ω scans	θ_max_ = 68.2°, θ_min_ = 4.8°
Absorption correction: multi-scan (*ABSCOR*; Higashi, 1995)	*h* = −8→8
*T*_min_ = 0.543, *T*_max_ = 0.912	*k* = −6→7
4753 measured reflections	*l* = −22→22
1566 independent reflections	

### Refinement

**Table:**

Refinement on *F*^2^	Secondary atom site location: difference Fourier map
*R*[*F*^2^ > 2σ(*F*^2^)] = 0.061	Hydrogen site location: inferred from neighbouring sites
*wR*(*F*^2^) = 0.175	H atoms treated by a mixture of independent and constrained refinement
*S* = 1.03	*w* = 1/[σ^2^(*F*_o_^2^) + (0.1029*P*)^2^ + 0.1188*P*] where *P* = (*F*_o_^2^ + 2*F*_c_^2^)/3
1566 reflections	(Δ/σ)_max_ < 0.001
126 parameters	Δρ_max_ = 0.49 e Å^−^^3^
0 restraints	Δρ_min_ = −0.39 e Å^−^^3^
Primary atom site location: structure-invariant direct methods	

### Special details

**Table:**

Geometry. All esds (except the esd in the dihedral angle between two l.s. planes) are estimated using the full covariance matrix. The cell esds are taken into account individually in the estimation of esds in distances, angles and torsion angles; correlations between esds in cell parameters are only used when they are defined by crystal symmetry. An approximate (isotropic) treatment of cell esds is used for estimating esds involving l.s. planes.
Refinement. Refinement was performed using all reflections. The weighted R-factor (wR) and goodness of fit (S) are based on F^2^. R-factor (gt) are based on F. The threshold expression of F^2^ > 2.0 sigma(F^2^) is used only for calculating R-factor (gt).

### Fractional atomic coordinates and isotropic or equivalent isotropic displacement parameters (Å^2^)

**Table:**

	*x*	*y*	*z*	*U*_iso_*/*U*_eq_	
F1	0.2762 (3)	−0.0078 (3)	0.52691 (8)	0.0597 (7)	
F2	0.3860 (2)	−0.3171 (3)	0.51696 (8)	0.0569 (6)	
F3	0.0979 (2)	−0.2730 (4)	0.50888 (9)	0.0661 (7)	
N4	0.3895 (3)	0.4055 (4)	0.31939 (13)	0.0343 (6)	
C5	0.3078 (3)	0.2521 (4)	0.27331 (13)	0.0272 (6)	
C6	0.2853 (3)	0.2886 (4)	0.19799 (12)	0.0293 (6)	
H6	0.3289	0.4171	0.1791	0.035*	
C7	0.2009 (3)	0.1398 (4)	0.15202 (13)	0.0311 (6)	
H7	0.1863	0.1670	0.1015	0.037*	
C8	0.1362 (4)	−0.0502 (4)	0.17775 (12)	0.0311 (6)	
H8	0.0788	−0.1528	0.1453	0.037*	
C9	0.1567 (3)	−0.0871 (4)	0.25119 (12)	0.0276 (6)	
H9	0.1123	−0.2164	0.2691	0.033*	
C10	0.2413 (3)	0.0608 (4)	0.30004 (11)	0.0220 (6)	
C11	0.2527 (3)	0.0253 (4)	0.37877 (12)	0.0276 (6)	
H11	0.2651	0.1475	0.4090	0.033*	
C12	0.2471 (3)	−0.1615 (4)	0.41076 (12)	0.0310 (6)	
H12	0.2410	−0.2856	0.3814	0.037*	
C13	0.2497 (4)	−0.1887 (4)	0.48945 (13)	0.0346 (7)	
H4A	0.448 (4)	0.362 (6)	0.3618 (18)	0.070 (11)*	
H4B	0.448 (5)	0.497 (5)	0.2944 (18)	0.067 (11)*	

### Atomic displacement parameters (Å^2^)

**Table:**

	*U*^11^	*U*^22^	*U*^33^	*U*^12^	*U*^13^	*U*^23^
F1	0.1045 (18)	0.0494 (12)	0.0245 (8)	0.0050 (11)	0.0032 (10)	−0.0071 (8)
F2	0.0673 (13)	0.0635 (13)	0.0370 (10)	0.0222 (10)	−0.0073 (9)	0.0154 (8)
F3	0.0571 (12)	0.1073 (17)	0.0329 (9)	−0.0274 (11)	−0.0002 (8)	0.0251 (10)
N4	0.0379 (14)	0.0297 (13)	0.0346 (13)	−0.0050 (11)	0.0003 (11)	−0.0022 (11)
C5	0.0267 (13)	0.0255 (13)	0.0292 (13)	0.0036 (11)	0.0021 (10)	−0.0008 (11)
C6	0.0319 (14)	0.0290 (14)	0.0271 (13)	0.0045 (12)	0.0035 (10)	0.0050 (11)
C7	0.0310 (14)	0.0389 (16)	0.0236 (12)	0.0041 (12)	0.0035 (10)	0.0037 (11)
C8	0.0332 (15)	0.0355 (15)	0.0239 (13)	−0.0023 (12)	0.0000 (11)	−0.0043 (11)
C9	0.0295 (13)	0.0276 (14)	0.0254 (12)	0.0026 (12)	0.0009 (10)	0.0004 (11)
C10	0.0211 (13)	0.0241 (13)	0.0201 (11)	0.0020 (10)	−0.0010 (9)	−0.0007 (10)
C11	0.0295 (15)	0.0293 (14)	0.0232 (12)	0.0010 (11)	−0.0008 (10)	−0.0024 (10)
C12	0.0388 (15)	0.0315 (15)	0.0218 (12)	0.0012 (12)	−0.0008 (11)	0.0002 (11)
C13	0.0400 (16)	0.0373 (16)	0.0254 (13)	0.0004 (13)	−0.0013 (11)	0.0035 (11)

### Geometric parameters (Å, º)

**Table:**

F1—C13	1.336 (3)	C7—H7	0.9500
F2—C13	1.348 (3)	C8—C9	1.378 (3)
F3—C13	1.326 (3)	C8—H8	0.9500
N4—C5	1.383 (3)	C9—C10	1.398 (3)
N4—H4A	0.90 (3)	C9—H9	0.9500
N4—H4B	0.88 (3)	C10—C11	1.475 (3)
C5—C10	1.409 (3)	C11—C12	1.318 (3)
C5—C6	1.412 (3)	C11—H11	0.9500
C6—C7	1.371 (3)	C12—C13	1.472 (3)
C6—H6	0.9500	C12—H12	0.9500
C7—C8	1.389 (4)		
			
C5—N4—H4A	118 (2)	C10—C9—H9	119.1
C5—N4—H4B	109 (2)	C9—C10—C5	119.0 (2)
H4A—N4—H4B	116 (3)	C9—C10—C11	121.2 (2)
N4—C5—C10	121.3 (2)	C5—C10—C11	119.7 (2)
N4—C5—C6	119.9 (2)	C12—C11—C10	125.6 (2)
C10—C5—C6	118.7 (2)	C12—C11—H11	117.2
C7—C6—C5	120.3 (2)	C10—C11—H11	117.2
C7—C6—H6	119.8	C11—C12—C13	123.7 (2)
C5—C6—H6	119.8	C11—C12—H12	118.1
C6—C7—C8	121.4 (2)	C13—C12—H12	118.1
C6—C7—H7	119.3	F3—C13—F1	106.1 (2)
C8—C7—H7	119.3	F3—C13—F2	106.1 (2)
C9—C8—C7	118.7 (2)	F1—C13—F2	104.4 (2)
C9—C8—H8	120.6	F3—C13—C12	113.4 (2)
C7—C8—H8	120.6	F1—C13—C12	113.9 (2)
C8—C9—C10	121.8 (2)	F2—C13—C12	112.1 (2)
C8—C9—H9	119.1		
			
N4—C5—C6—C7	−178.5 (2)	N4—C5—C10—C11	2.2 (4)
C10—C5—C6—C7	−0.3 (4)	C6—C5—C10—C11	−176.1 (2)
C5—C6—C7—C8	−0.3 (4)	C9—C10—C11—C12	24.6 (4)
C6—C7—C8—C9	0.5 (4)	C5—C10—C11—C12	−158.9 (3)
C7—C8—C9—C10	−0.3 (4)	C10—C11—C12—C13	−176.8 (2)
C8—C9—C10—C5	−0.2 (4)	C11—C12—C13—F3	115.4 (3)
C8—C9—C10—C11	176.3 (2)	C11—C12—C13—F1	−6.2 (4)
N4—C5—C10—C9	178.7 (2)	C11—C12—C13—F2	−124.5 (3)
C6—C5—C10—C9	0.5 (3)		

### Hydrogen-bond geometry (Å, º)

*Cg*1 is the centroid of the C5–C10 ring.

**Table:**

*D*—H···*A*	*D*—H	H···*A*	*D*···*A*	*D*—H···*A*
N4—H4*A*···F2^i^	0.90 (3)	2.46 (4)	3.352 (3)	169 (3)
C12—H12···N4^ii^	0.95	2.56	3.432 (4)	152
N4—H4*B*···*Cg*1^iii^	0.88 (3)	2.59 (4)	3.315 (2)	140 (3)
C9—H9···*Cg*1^iv^	0.95	2.73	3.480 (3)	136

Symmetry codes: (i) −*x*+1, −*y*, −*z*+1; (ii) *x*, *y*−1, *z*; (iii) −*x*+1, *y*+1/2, −*z*+1/2; (iv) −*x*, *y*−1/2, −*z*+1/2.
